# Functions of Exosomes in the Triangular Relationship between the Tumor, Inflammation, and Immunity in the Tumor Microenvironment

**DOI:** 10.1155/2019/4197829

**Published:** 2019-08-01

**Authors:** Tiantian Wang, Moussa Ide Nasser, Jie Shen, Shujuan Qu, Qingnan He, Mingyi Zhao

**Affiliations:** Department of Pediatrics, The Third Xiangya Hospital, Central South University, Changsha, Hunan Province 410013, China

## Abstract

Exosomes are extracellular vesicles that contain diverse components such as genetic materials, proteins, and lipids. Owing to their distinct derivation and tissue specificity, exosomes act as double-edged swords during the development of neoplasms. On the one hand, tumor-derived exosomes can modulate the immune system during tumorigenesis by regulating inflammatory cell infiltration and oxidative stress and by promoting epithelial-to-mesenchymal transition and immune-induced tumor dormancy. On the other hand, components of specific immune cell-derived exosomes may contribute to the efficacy of antitumor immunotherapy. In this review, we demonstrate the pivotal role of exosomes in the triangular relationship in the tumor microenvironment between the tumor, inflammation, and immunity, which may provide potential strategies for tumor immunotherapy at genetic and cellular levels.

## 1. Introduction

Exosomes are vesicles that contain genetic materials, lipids, and functional proteins, and exosomes secreted by cancer and immune cells contain cell-specific content. Owing to their widespread and stable existence in biological fluids, exosomes may act as useful biomarkers for detecting the progression of cancers [1]. Intriguingly, exosomes exert bidirectional effects on cancers as a result of their distinct origins and heterogeneity. For example, some tumor-derived exosomes (TDEs) act as tumor growth stimulators, activating the epithelial-to-mesenchymal transition (EMT) and tumor dormancy during cancer proliferation, invasion, and metastasis, whereas other exosomes that originate from specific immune cells act as inhibitors that interfere with cancer growth [2]. In addition, many studies have demonstrated the significance of inflammation in the initiation and development of tumors, and it has been shown that exosomes can affect the progression of inflammation in the tumor environment by initiating inflammatory pathways, activating neutrophils, and regulating oxidative stress. This review discusses the latest research on the functions of exosomes in the triangular relationship between the tumor, inflammation, and immunity in the tumor environment and provides a basis for the potential use of exosomes as vectors in tumor gene therapy and tumor immunotherapy.

## 2. Exosomes

### 2.1. Exosomes and Their Biological Characteristics

Extracellular vesicles (EVs) consist of three main subtypes based on their biogenesis: exosomes, microvesicles, and apoptotic bodies [3, 4]. Exosomes are vesicles 40–200 nm in diameter marked by tetraspanins, Alix, and TSG101. Microvesicles are around 200-2000 nm in size and are marked by integrins, selectins, and CD40. Apoptotic bodies are around 500-2000 nm in size and are marked by phosphatidylserines and genomic DNA [5]. Exosomes, which were first identified in sheep reticulocytes in 1985 [6], are double-layered lipid membrane-enclosed vesicles that are secreted by almost all viable cells under both normal and pathological conditions and are extensively present in body fluids, intercellular spaces, and tissues [7]. Exosomes contain diverse proteins, lipids, and nucleic acids, such as microRNAs (miRNAs), long noncoding RNAs (lncRNAs), and circular RNAs (circRNAs). There are two types of proteins present in exosomes. The first type, which exists in most exosomes and can function as a marker, includes heat-shock proteins (HSPs), transmembrane 4 superfamily proteins, membrane transport proteins, and fusion proteins [8]. The second type, which is cell-specific and has heterogeneous functions, includes major histocompatibility complex II (MHCII) and fas ligand (FasL), which are present in exosomes from lymphoblastoid cells and induce apoptosis in CD4^+^ T cells [9]. Exosomes are rich in cholesterol, glycosphingolipids, ether lipids, and phosphatidylserine, which participate in both biogenesis and structural maintenance [10–12]. The first release of the exosome database included 58,330 circRNAs, 15,501 lncRNAs, and 18,333 mRNAs, which suggests a sophisticated genetic control system [13]. These functional RNAs can affect biological activities and modulate cellular events such as cell proliferation, apoptosis, differentiation, and immunoregulation [14–16].

### 2.2. Exosomes and Intercellular Communication

Exosomes participate in intercellular communication. Tumor-derived exosomal lncRNAs have been implicated as signaling mediators that coordinate the functions of neighboring tumor cells. Interestingly, some exosomal RNAs from donor cells can function in recipient cells and are called “exosomal shuttle RNAs,” suggesting a role in genetic exchange between cells [17]. For example, after stimulation with arsenite, exosomes derived from hepatic epithelial cells can transfer circRNA_100284 to surrounding cells, which increases the expression of enhancer zeste homolog 2 (EZH2) and cyclin-D1 and subsequently promotes the G1/S transition [18]. This finding demonstrates the oncogenic capacity of exosomes. Through intercellular communication, changes in an individual cell may influence the course of tumor proliferation and metastasis on a macroscale. In fact, in epithelial ovarian cancer, tumor-secreted exosomes transfer miR-99a-5p to adjacent human peritoneal mesothelial cells (HPMCs), resulting in increased levels of fibronectin and vitronectin, extracellular matrix components that are closely associated with tumor invasion [19].

### 2.3. Exosomes and Inflammation

Another vital function of exosomes that is related to disease progression is the modulation of inflammation [20, 21]. Exosomes can promote or inhibit the development of inflammation. Hypoxia-induced delivery of miR-23a from exosomes secreted by tubular epithelial cells was shown to promote macrophage activation and trigger tubulointerstitial inflammation [22]. Similarly, miR-150-5p and miR-142-3p from dendritic cell- (DC-) released exosomes can be transferred to regulatory T cells (Tregs), resulting in an increase in interleukin 10 (IL-10) expression and a decrease in IL-6 expression [23]. Choroid plexus epithelial cells can release exosomes that contain miR-146a and miR-155, which upregulate the expression of inflammatory cytokines in astrocytes and microglia [24]. Another type of exosome exhibits protective effects against inflammation-related diseases [25]. In endometriosis, exosomal miR-138 can protect against inflammation by decreasing the expression level of nuclear factor-*κ*B (NF-*κ*B), a transcription factor that regulates inflammatory cytokines, such as tumor necrosis factor-*α* (TNF-*α*) and IL-18 [26]. In addition, a study showed that exosomes secreted by bone marrow mesenchymal stem cells (BMSCs) can attenuate inflammatory changes in a rat model of experimental autoimmune encephalomyelitis by modulating microglial polarization and maintaining the balance between M2-related and M1-related cytokines [27]. Another study revealed that exosomal miR-181c suppressed Toll-like receptor 4 (TLR-4) expression and subsequently lowered TNF-*α* and IL-1*β* levels in burn-induced inflammation [28]. Treg-derived exosomes containing miR-Let-7d affected T helper cell 1 (Th1) cell growth and inhibited IFN-*γ* secretion to inhibit inflammation [29]. Exosomal miR-155 from bone marrow cells (BMCs) was shown to enhance the innate immune response in chronic inflammation by increasing TNF-*α* levels [30]. These findings provide a basis for investigating the role of inflammation in the tumor microenvironment, as well as the possibility of utilizing exosomes as a carrier to attenuate inflammation and restore impaired immune responses in cancer.

## 3. The Function of Exosomes in the Tumor Microenvironment and Metabolism

### 3.1. Exosomes Are Involved in Immune Activities during Tumorigenesis

**Figure 1:**
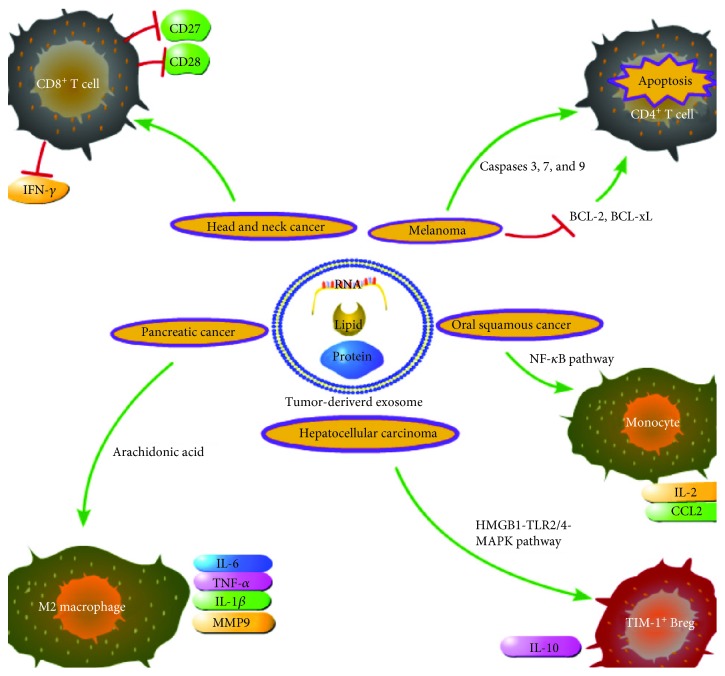
Various effects of tumor-derived exosomes on the immune system and possible associated pathways in the tumor microenvironment.

Tumor occurrence is strongly correlated with a failure in immune surveillance, and surprisingly, the translocation of tumor-derived exosomes may assist in immune escape by interfering with cellular events, such as immune cell differentiation and cytokine secretion. One study showed that exogenous circRNAs activate the expression of retinoic-acid-inducible gene-I (*RIG-I*) and initiate innate immunity [31]. This study clearly demonstrated that foreign genes could affect the endogenous genes in cells and induce an immune reaction. Zhou et al. showed that the melanoma-derived exosomal miRNA-Rab27a could be taken up by CD4^+^ T cells and may accelerate mitochondrial apoptosis and upregulate the expression of B-cell lymphoma-2 (BCL-2) and B-cell lymphoma-extra large (BCL-xL), which are antiapoptotic proteins [32] (Figure 1). This result demonstrates the interaction between tumor cells and immune cells. This finding is not unique, and other studies have reported similar results. For example, Ning et al. showed that exosomes from lung carcinoma or breast cancer cells could block the differentiation of CD4^+^IFN-*γ*^+^ Th1 cells, inhibit the maturation and migration of DCs, and induce apoptosis to promote the immunosuppressive effect of DCs [33]. Head and neck cancer cell- (HNCC-) derived exosomes affect CD8^+^ T cells by inducing a loss of CD27/CD28, accompanied by decreased levels of the antitumor cytokine IFN-*γ* [34] (Figure 1). miR-29a-3p and miR-21-5p in tumor-related macrophage-derived exosomes inhibit the STAT3 signaling pathway and increase the Treg/T helper cell 17 (Th17) ratio in epithelial ovarian cancer, which may lead to poor patient outcomes [35]. Human hepatocellular carcinoma- (HCC-) derived exosomes containing high mobility group box 1 (HMGB1) promote the proliferation of the T cell immunoglobulin domain and mucin domain protein-1 positive regulatory B (TIM-1^+^ Breg) cells by inducing the Toll-like receptor/mitogen-activated protein kinase (TLR/MAPK) pathway and the production of IL-10, an immunosuppressive cytokine, thus working against CD8^+^ T cells through the TLR2/4-MAPK pathway and leading to immune surveillance failure [36] (Figure 1). These findings demonstrate that TDEs are involved in generating an immunosuppressive microenvironment that promotes the progression of solid tumors. In addition, exosomes may also promote tumor progression in hematological cancers. For example, elevated levels of the lncRNA HOX transcript antisense (HOTAIR) can lead to decreased T-lymphocyte proliferation as well as reduced immunoglobulin production and a reversed ratio of CD4^+^/CD8^+^ T cell subsets through the Wnt/*β*-catenin pathway, which may eliminate the immunologic rejection of leukemia cells [37]. Exosomal circRNAs from tumor cells play a crucial role in poor prognosis in cancer [38]; however, few studies have examined their interaction with the immune system. The immunosuppressive role of tumor-secreted exosomes suggests their potential in tumor therapy, as blockage of tumor-secreted exosomes may enhance the antitumor effects of tumor-related T cells and inhibit the silencing of these cells [39]. However, specific immune cell-derived exosomes may also be conducive to tumor development. In colorectal cancer, macrophage-derived exosomes containing miR-21-5p and miR-155-5p can regulate the Brahma-related gene 1 (*BRG1*) coding sequence to promote the metastasis of cancer cells (Table 1) [40].

### 3.2. Exosomes, Oxidative Stress, and Inflammation during Tumor Progression

A sustained oxidative stress state may trigger chronic inflammation through activation of inflammatory pathways [41]. During chronic inflammation, slow release of reactive oxygen species (ROS) can cause genetic mutations in nearby cells, promote the proliferation of malignantly transformed cells, and inhibit apoptosis [41, 42]. For example, pancreatic cancer cell-derived exosomes have been shown to regulate STAT3 signaling in monocytes and induce the expression of arginase and ROS [43]. In contrast, leukemic cell-derived exosomes increase the levels of inflammatory mediators such as TNF-*α* and IL-10 in macrophages but decrease ROS levels in BMSCs, thus turning the local bone marrow into a leukemia-friendly microenvironment [44]. These results reflect the different ROS states in solid and nonsolid tumors.

Chronic inflammation can initiate cellular activities that contribute to the malignant transformation of cells, particularly DNA damage and genetic instability [45, 46]. TDEs may accelerate tissue damage and inflammation during tumor progression. In pancreatic cancer, macrophages treated with TDEs secreted a greater amount of inflammatory molecules, including IL-6, IL-1*β*, and TNF-*α* [47] (Figure 1). Additionally, in oral cancer, monocytes can take up extracellular vesicles, which promote the activation of NF-*κ*B and the establishment of a proinflammatory milieu marked by increased levels of IL-6 and matrix metallopeptidase 9 (MMP9) [48] (Figure 1). HCC cell-derived exosomes containing abundant *lncRNA TUC339* cause a reduction in the levels of proinflammatory mediators, expression of costimulatory molecules, and phagocytosis activity in macrophages [49], resulting in the inhibition of immune activity. Colon cancer cells secrete exosomes containing *miR-1246* that can reprogram macrophages to promote the generation of an anti-inflammatory environment via increased expression of TGF-*β* [50]. Additionally, neutrophils, a signature of inflammation, can regulate immunosuppression to promote tumor progression via suppression of natural killer cell activity [51]. Zhang et al. showed that gastric cancer cell-derived exosomes increase the number of inflammatory factors and activate neutrophils in an HMGB1/TLR4/NF-*κ*B axis-dependent manner, which can promote tumor metastasis [52].

Nevertheless, exosomes can also be ideal tools to slow tumor progression. Mao et al. reported that exosomes could carry *esophageal cancer-related gene-4* (*Ecrg-4*) mRNA and inhibit the expression of genes related to angiogenesis and inflammation [53]. Interestingly, camel milk-derived exosomes can slow the development of breast cancer by inducing tumor cell apoptosis, reducing oxidative stress and the release of inflammatory cytokines, and activating the immune response by increasing the numbers of CD 4^+^ and CD8^+^ T cells (Table 1) [54].

### 3.3. Exosomes, Inflammation, and Immunity in the Tumor Microenvironment

Chronic inflammation may act as a negative mediator in tumor immunity through myeloid-derived suppressor cells (MDSCs), which are precursors of immune cells such as macrophages, DCs, and granulocytes. A recent report showed that increased levels of the inflammatory cytokine IFN-*γ* can disrupt the differentiation of MDSCs and thus interfere with antigen presentation in tumors [55]. TDEs may be involved in this process of immune suppression. In a mouse model of glioma, TDEs containing *miR-10a and miR-21* can be engulfed by MDSCs and affect their development to produce immunosuppressive molecules [56]. Similarly, mast cells (MCs) can be considered the bridge between inflammation and immunity in the tumor and may participate in angiogenesis and lymphangiogenesis [57]. The function of TDEs on mast cells is not clear and requires further investigation. On the other hand, lung cancer cells can take up MC-derived exosomes containing the protein KIT and attain rapid growth [58]. In this way, MC-derived exosomes could be a key target for tumor immunotherapy. One report has shown that exosomes from mast cells processed by hepatitis C virus E2 (HCV-E2) can block HCC metastasis [59].

### 3.4. Exosomes and Immune Tolerance

It has been shown that the lncRNA-SNHG14 can enhance the efficiency of trastuzumab in breast cancer by targeting the B-cell lymphoma-2/B-cell lymphoma-2 associated X (Bcl-2/Bax) signaling pathway, which regulates apoptosis [60]. In esophageal squamous cell carcinoma (ESCC), elevated levels of exosomal lncRNA *prostate androgen-regulated transcript 1* (*PART1*) caused resistance to gefitinib by binding to miR-129 and increasing the expression of Bcl-2 [61]. Similarly, for melanoma patients with no reaction to immunization therapy, exosomal PD-L1 derived from melanoma cells was shown to interfere with the antitumor activity of immune cells by binding to CD8^+^ T cells, inhibiting the proliferation of tumor-infiltrating CD8^+^ T lymphocytes (TILs) and reducing the production of IFN-*γ* and IL-2 [62]. Kanlikilicer et al. showed that exosomes from paclitaxel-resistant ovarian cancer (OC) cells could transfer miR-1246 to M2-type macrophages, allowing miR-1246 to bind to the 3′UTR of caveolin-1 (*Cav1*) and function through platelet-derived growth factor receptor (PDGFR) tyrosine signaling to inhibit cell proliferation [63]. Amazingly, exosomes could be used as a tool to overcome the problem of drug resistance. A silencing RNA- (siRNA-) targeting GRP78 (siGRP78) contained in exosomes from BMCs was able to impede tumor cell proliferation, invasion, and metastasis in HCC [64]. Gene silencing could also be applied to other chemotherapy-tolerant cancers, as long as the genetic mechanism underlying the effects of the exosomes is known.

### 3.5. Exosomes and EMT

EMT initiates the conversion of malignant tumor epithelial cells to an interstitial phenotype, which can promote invasion and metastasis. A recent study showed that BMSCs in a hypoxic state transferred exosomal-derived miR-193a-3p, miR-210-3p, and miR-5100 to activate signal transducer and activator of transcription 3 (STAT3) signaling in lung cancer cells and also led to an increase in the levels of vimentin and N-cadherin, two mesenchymal markers [65]. These results strongly suggest that TDEs are involved in tumor EMT progression. Following pretreatment with TGF-*β*, an inflammatory cytokine, exosomal-derived *lnc-MMP2-2* was shown to promote the expression of matrix metalloproteinase-2 (MMP2), an important EMT marker, to regulate the dissemination of lung cancer cells through the vasculature [66]. This demonstrates the participation of the immune system in EMT. Furthermore, *Snail*, an EMT transcriptional factor, was shown to activate the M1 macrophage- (M1 M*Ф*-) M2 macrophage (M2 M*Ф*) transition by increasing TDEs-miR-21 and transferring TDEs to CD14^+^ human monocytes, which promotes the advancement of HNC [67]. Therefore, the import of exosomal siRNAs into tumor cells may be an effective tumor gene and immunotherapy method to suppress the expression of target mRNAs [68]. Interestingly, the impact of exosomes on EMT is not completely one-sided. For example, exosomes containing miR-128-3p block EMT by inhibiting the mRNA expression of B-cell-specific Moloney murine leukemia virus integration site 1 (*Bmi1*) in CRC [69]. Additionally, cancer-associated fibroblasts (CAFs) can secrete exosomes lacking miR-148b that are transferred to endometrial cancer cells (ECCs) and modulate EMT by relieving the suppression of DNA (cytosine-5)-methyltransferase 1 (*DNMT1*) [70]. *miR-155-5p* in exosomes derived from gastric cancer cells induced a mesenchymal-like morphological change and increased the levels of E-cadherin and vimentin as well as resistance to paclitaxel, a classical chemotherapy medicine [71]. These studies demonstrate the significance of exosomes in the modulation of gene expression, components of the extracellular matrix, basement membrane remodeling, and tumor chemotherapy (Table 2).

### 3.6. Exosomes and Tumor Dormancy

Immune-induced tumor dormancy refers to the phenomenon of cell cycle arrest, downregulation of proliferation-related genes, and slowing of metabolism in tumor cells, which is regulated by the immune system. This quiescent state may be reversed through interactions between exosomes and tumor cells. It has been reported that miR-93 and miR-193 act to decrease cyclin D1 and induce quiescence in glioblastoma multiforme (GBM), which leads to a lower percentage of cycling cells [72]. In addition, increased levels of miR-23b in breast cancer cells transferred from exosomes suppressed the expression of *MARCKS*, which encodes a protein that facilitates cell cycling [73]. However, in the bone marrow of bladder cancer patients, M1 M*Ф*-derived exosomes may convert quiescent tumor cells into cycling cells through NF-*κ*B, while M2 M*Φ*-derived exosomes may contribute to dormancy [74]. This finding exhibits the diverse immunoregulatory roles of immune cells during tumor progression (Table 2).

## 4. Exosomes in Tumor Immunotherapy

### 4.1. Exosomes and the Immune Checkpoint Protein PD-1

Programmed death-1 (PD-1), which is expressed on the surface of immune cells, and its ligand programmed cell death-1 ligand 1 (PD-L1), which is expressed in various tumor tissues, are significant immunosuppressive molecules, and their interaction can induce T cell apoptosis and inhibit T cell proliferation, promoting tumor progression. Therefore, inhibitors of PD-1 and PD-L1 are ideal antitumor immune sentinel-related drugs. Recent studies have revealed that exosomes may participate in PD-1-related anti- or protumorigenesis effects. The main obstacle to successful immunotherapy is immunosuppression, and the accumulation of MDSCs is the main mechanism of immunosuppression. It has been reported that oral squamous cell carcinoma- (OSCC-) derived exosomes can regulate MDSCs through the miR-21/PTEN/PD-L1 pathway and suppress the cytotoxicity of *γδ* T cells [75]. Chronic lymphocytic leukemia- (CLL-) derived exosomes containing the noncoding Y RNA hY4 can turn monocytes into procarcinogenic cells through Toll-like receptor 7 (TLR7) signaling, and these cells may release tumor-related cytokines such as C-C motif chemokine ligand 2 (CCL2), CCL4, and IL-6 to generate an inflammatory environment and increase the expression of PD-L1 on the surface of tumor cells to induce immune escape [76]. In addition, exosomes can alter the tumor microenvironment by enveloping PD-L1. Theodoraki et al. showed that PD-L1(+) exosomes in the plasma of patients with HNSCC could inhibit the expression of CD69 on CD8^+^ T cells, which is a signature of activated T cells [77]. In immunotherapy, treatment with dendritic cells pulsed with TDEs in combination with the PD-1 antibody was shown to enhance the effect of sorafenib, leading to an increased number of PD-1^+^ CD8^+^ T cells, and was more efficient than sorafenib alone [78]. In addition, plasma-derived exosomes containing PD-L1 mRNA may enhance the efficiency of nivolumab and pembrolizumab for the treatment of melanoma and non-small cell lung cancer (NSCLC) [79]. *γδ*T cell-derived exosomes containing miR-138 have potential as a drug delivery system targeting PD-1 and CTLA-4 in CD8^+^ T cells to increase their cytotoxicity in OSCC [80]. However, more evidence for the feasibility of TDE use for clinical applications is needed (Table 3).

### 4.2. Exosomes and Adoptive Cell Transfer

Adoptive cell transfer (ACT) therapy uses effector cells and immune molecules to directly attack tumor cells and is called “passive” immunotherapy. One of the main players in ACT therapy is tumor-infiltrating lymphocytes (TILs). TILs are a group of heterogeneous antitumor lymphocytes present in tumor tissues that include CD8^+^ T cells, some CD4^+^ T cells, a small number of B cells, NK cells, macrophages, DCs, MDSCs, and Tregs. Recent studies have shown that exosomes are involved in TIL-related immunotherapy. For example, exosomal 14-3-3 protein zeta (14-3-3*ζ*) shed from HCC cells can be transferred to TILs and interfere with their antitumorigenesis function [81]. In contrast, Li et al. showed that exosomes derived from DCs can boost the proliferation of naive T cells, subsequently increase the number of cytotoxic T lymphocytes (CTLs), and initiate an immune reaction against HCC [82]. In addition, exosomes released by NK cells can function as fuel for the immune killing machines against various tumors, including glioblastoma, melanoma, and other cancers, in a TNF-*α*- and granzyme B-related manner [83–85]. NK cell-derived exosomes containing miR-186 exert their cytotoxic effects against neuroblastoma by inhibiting the expression of *MYCN* and *TGFBR1* [86]. These findings suggest that immune cell-derived exosomes may contribute to immune activation against tumors by functioning as unmanned vehicles (Table 3).

## 5. Summary and Perspectives

Exosomes are attracting increasing interest owing to their significant heterogeneity and their ability to regulate the tumor immune microenvironment. Specifically, tumor cell-derived exosomes may accelerate tumor progression by enhancing immunosuppression and inflammation, increasing oxidative stress, inducing EMT, and regulating tumor dormancy, which may lead to a poor prognosis. In contrast, specific immune cell-derived exosomes can act as tumor inhibitors, suggesting their immense potential for use in cancer immunotherapy. However, various possible strategies for their use remain to be validated. At the gene level, some ncRNAs, including miRNAs, circRNAs, and lncRNAs, have been suggested to be closely associated with carcinogenesis. Therefore, if an siRNA can be transferred to tumors via exosomes, it may be able to subsequently downregulate target mRNA expression and inhibit tumor invasion [87]. From the perspective of cell therapy, the interactions between immune cells and tumor cells via exosomes may allow us to modulate immune reactions against cancers. For example, overexpression of protective contents in immune cell-secreted exosomes may assist in killing the tumor cell. In addition, exosomes can be used as carriers for gene therapy and immune therapy to deliver specific tumor-related molecules, such as PD-1 [88]. However, one obstacle preventing the widespread use of exosomes in clinical practice is that the yield of exosomes from traditional culture is low. Additionally, the methods used for the separation and purification of exosomes, namely, ultracentrifugation and sucrose density gradient centrifugation, are time-consuming and laborious. Further studies on exosomes and modifications are needed to solve these problems and facilitate the clinical application of exosomes as drug carriers in antitumor immunotherapy.

## Figures and Tables

**Table 1:** Exosome functions in the tumor microenvironment: effects on the tumor, immunity, and inflammation.

Tumor type	Exosome content	Exosome origin	Effector cells	Effector components
Melanoma	miR-Rab27a	Tumor	CD4^+^ T cells	BCL-2, BCL-xL
LC, breast cancer	/	Tumor	CD4^+^IFN-*γ*^+^ Th1 cells, DCs	/
HNC	/	Tumor	CD8^+^ T cells	CD27/CD28, IFN-*γ*
EOC	miR-29a-3p, miR-21-5p	Macrophage	Treg/Th17 cells	STAT3 signaling, Treg/Th17 ratios
HCC	*HMGB1*	Tumor	T cells, TIM-1^+^ Breg cells, CD8^+^ T cells	TLR/MAPK pathway, IL-10
Leukemia	lncRNA HOTAIR	Tumor	T lymphocytes, CD4^+^/CD8^+^ T cells	Wnt/*β*-catenin axis, Ig
CRC	miR-21-5p, miR-155-5p	Macrophage	Tumor cells	*BRG1*
PC	/	Tumor	Macrophages	IL-6, IL-1*β*, and TNF-*α*
OC	/	Tumor	Monocytes	NF-*κ*B pathway, IL-6, MMP9
HCC	lncRNA TUC339	Tumor	Macrophages	Costimulatory molecules
CC	miR-1246	Tumor	Macrophages	TGF-*β*
GC	/	Tumor	Neutrophils	HMGB1/TLR4/NF-*κ*B
PC	/	Tumor	Monocytes	STAT3 signaling, arginase, ROS
Leukemia	/	Tumor	Macrophages, BM-MSCs	TNF-*α*, IL-10, ROS
EC	*Ecrg-4* mRNA	Tumor	/	Inflammation-related genes
Breast cancer	/	Camel milk	Tumor cells, CD 4^+^ T cells, CD8^+^ T cells	Tumor cell apoptosis, ROS
Glioma	miR-10a, miR-21	Tumor	MDSCs	ROS, IL-10, TGF-*β*
LC	KIT	Mast cell	Tumor cells	KIT/SCF pathway

Tumor abbreviations: LC: lung cancer; HNC: head and neck cancer; EOC: epithelial ovarian cancer; CRC: colorectal cancer; EC: esophageal cancer; PC: pancreatic cancer; OC: oral cancer; CC: colon cancer; and GC: gastric cancer. “/” means “not mentioned”.

**Table 2:** Exosomes, EMT, and tumor dormancy.

Function	Tumor	Exosome contents	Exosome origin	Effector
EMT	LC	miR-193a-3p, miR-210-3p, miR-5100	BMSCs	STAT3 signaling, vimentin, N-cadherin
	LC	lnc-MMP2-2	/	MMP2
	HNC	miR-21	Tumor	Snail, CD14^+^ human monocytes
	CRC	miR-128-3p	/	Bmi1/E-cadherin, MRP5
	EC	Lacking miR-148b	CAFs	*DNMT1*
	GC	miR-155-5p	Tumor	E-cadherin, vimentin
Tumor dormancy	GBM	miR-93, miR-193	Tumor	Cyclin D1
	Bladder cancer	/	M1 M*Ф*s	NF-*κ*B p65
	Breast cancer	miR-23b	BMSCs	*MARCKS*

Tumor-type abbreviations: LC: lung cancer; HNC: head and neck cancer; EC: endometrial cancer; CRC: colorectal cancer; GBM: glioblastoma multiforme; GC: gastric cancer. “/” means “not mentioned”.

**Table 3:** Exosomes and immunotherapy.

Tumor type	Exosome contents	Exosome origin	Effector cells	Active components
OSCC	miR-21	Tumor	MDSCs	PTEN/PD-L1
CLL	Y RNA hY4	Tumor	Monocytes	TLR7/CCL2, CCL4, IL-6
HNSCC	PD-L1(+)	Tumor	CD8^+^ T cells	CD69
HCC	PD-1 antibody	Tumor	DCs	PD-1^+^ CD8^+^ T cells
	miR-138	*γδ* T cell	CD8^+^ T cells	PD-1, CTLA-4
	14-3-3*ζ*	Tumor	TILs	Cell proliferation
	/	DCs	Naive T cells, CTLs	Cell proliferation
Glioblastoma melanoma	/	NK cells	Tumor cells	TNF-*α*, granzyme B
Neuroblastoma	miR-186	NK cells	Tumor cells	*MYCN*, *TGFBR1*

Tumor-type abbreviations: OSCC: oral squamous cell carcinoma; HCC: hepatocellular carcinoma; HNSCC: head and neck squamous cell carcinoma; CLL: chronic lymphocytic leukemia. “/” means “not mentioned”.
